# MicroRNAs as Potential Biomarkers for Alzheimer’s Disease in Women

**DOI:** 10.3390/genes16080943

**Published:** 2025-08-11

**Authors:** Shiwei Huang, Lily Zhong, Lilly Zheng, Jian Shi

**Affiliations:** 1 Department of Neurosurgery, University of Minnesota, Minneapolis, MN 55455, USA; 2 Department of Neurology, Department of Veterans Affairs Health Care System, San Francisco and University of California, San Francisco, CA 94121, USA

**Keywords:** Alzheimer’s disease, sex differences, miRNAs, biomarkers, inflammation, female specific mechanism

## Abstract

Alzheimer’s disease (AD) affects approximately 50 million people worldwide, with women comprising two-thirds of those affected. Despite this disproportionate impact, the sex-specific pathological mechanisms underlying AD in women remain poorly understood, and female-specific biomarkers have been significantly understudied. This critical knowledge gap requires focused research to improve diagnostic and therapeutic approaches for women with AD. In this review, we systematically examine the pathological mechanisms underlying AD in women, including sex-related differences in inflammation, autophagy, and metabolic dysfunction. We further explore microRNA (miRNA) expression patterns and evaluate miRNA candidates as potential biomarkers for AD in women based on current literature. Through this analysis, we identified approximately 20 miRNA candidates derived from diverse human samples, including brain tissue, blood, and cerebrospinal fluid, in multiple independent studies. These candidates demonstrate the potential for developing accessible, non-invasive biomarkers, particularly those identified in blood and cerebrospinal fluid. However, the limited overlap between studies highlights that female-specific miRNA biomarker research for AD remains in its early discovery phase, emphasizing the urgent need for large-scale validation studies and standardized methodological approaches to advance this promising field for clinical application.

## 1. Introduction

Alzheimer’s disease (AD) is the leading cause of dementia, affecting approximately 50 million people worldwide [1]. The hallmark neuropathological features of AD include β-amyloid (Aβ) plaques, neurofibrillary tangles composed of hyperphosphorylated tau proteins, and progressive neurodegeneration, which collectively distinguish AD from other forms of dementia. A striking epidemiological feature of AD is its disproportionate impact on women, who comprise more than 65% of patients with AD and face approximately twice the lifetime risk of developing AD compared to men (20% versus 10%) [2]. While women’s longer lifespan partially explains this disparity, emerging evidence suggests that biological sex differences play a more fundamental role in AD pathogenesis. Women with AD exhibit more severe symptoms, more rapid cognitive decline, neuropathology, and more cognitive deterioration than men [3]. Although there are two primary and apparent mechanisms that underlie these sex differences, the direct effects of sex hormones, particularly estrogen and progesterone, and the indirect effects of sex chromosomes, especially X-linked genes that exhibit disproportionate sex-specific expression variance [4], AD in women has been understudied and not diagnosed and treated precisely, especially due to the lack of non-invasive and effective biomarkers to detect and prevent the disease in its early stages.

MicroRNAs (miRNAs) have emerged as stable and non-invasive biomarker candidates in blood samples, with altered expression patterns in blood, serum, and cerebrospinal fluid serving as potential diagnostic and prognostic indicators for neurological disorders [5,6]. miRNAs are small non-coding RNAs that regulate gene expression post-transcriptionally and play crucial roles in AD pathogenesis, particularly in tauopathies that are more relevant to female patients with AD [7]. AD can be broadly classified into three different stages: mild cognitive impairment (MCI), moderate, and severe stages, based on the progression of the disease. MCI is a prodromal phase that does not invariably progress to AD, thus highlighting the importance of early diagnosis and treatment for optimal outcomes. However, sex-specific miRNA expression patterns in AD remain understudied, indicating a critical gap in knowledge.

This review examines the theoretical foundations and empirical evidence supporting the use of miRNAs as biomarkers for detecting and predicting AD in women. We explore sex differences in key biological processes—inflammation, metabolism, and autophagy—and their associated miRNA regulatory networks in AD pathogenesis, aiming to identify miRNA candidates specific to women with AD for early detection and therapeutic intervention.

## 2. Search Strategy and Inclusion Criteria

Database and Search Terms: We conducted a comprehensive literature search using PubMed as the primary database. The search strategy employed multiple keyword combinations to ensure thorough coverage: (1) “Gender OR Sex” AND “miRNA” AND “Alzheimer’s disease OR AD”; (2) “Female” AND “miRNA” AND “Alzheimer’s disease”; and targeted searches combining gender/sex terms with specific mechanisms: (3) “Gender OR Sex” AND “miRNA” AND “Inflammation” AND “Alzheimer’s disease”; (4) “Gender OR Sex” AND “miRNA” AND “Autophagy” AND “Alzheimer’s disease”; (5) “Gender OR Sex” AND “miRNA” AND “Metabolism” AND “Alzheimer’s disease”; and (6) “Gender OR Sex” AND “miRNA” AND “Biomarkers” AND “Alzheimer’s disease.”

Inclusion Criteria: Studies were included if they (1) investigated the diagnostic potential of miRNAs in human Alzheimer’s disease or (2) examined miRNA-related AD mechanisms in either patients with AD or animal models. No language or publication date restrictions were imposed.

Study Selection: This narrative review synthesizes the existing literature without generating new experimental data, focusing on gender-specific aspects of miRNA research in Alzheimer’s disease.

## 3. Gender Differences Lead to Biological Mechanical Differences in Diseases

Sex differences, mainly mediated by sex hormones and X chromosomes, contribute to distinct biological mechanisms in aging and disease, particularly in inflammation, metabolism, and autophagy. These mechanistic differences help explain the disproportionate impact of Alzheimer’s disease on women and provide a foundation for understanding sex-specific miRNA-regulatory networks.

### 3.1. Inflammation and Immune Cell Responses

**Figure 1:**
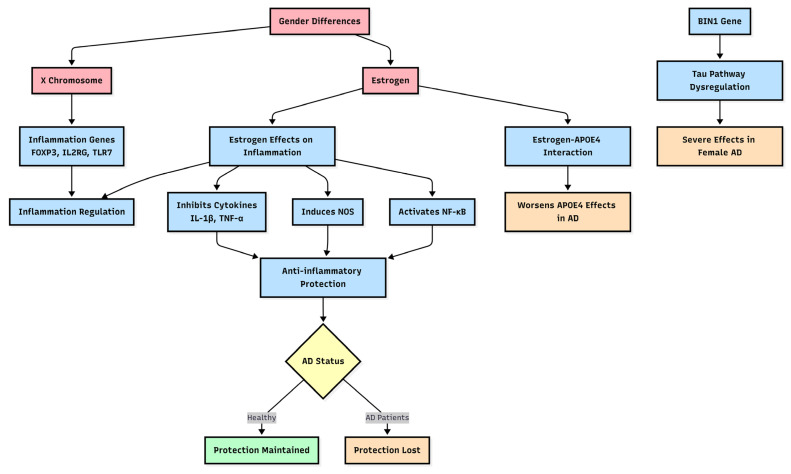
Gender Differences in Inflammation Pathways in AD. All abbreviations used in the diagram include: AD (Alzheimer’s disease), *FOXP3* (Forkhead box P3), *IL2RG* (Interleukin 2 receptor subunit gamma), TLR7 (Toll-like receptor 7), *IL-1β* (Interleukin 1 beta), *TNF-ά* (Tumor necrosis factor-alpha), *NOS* (Nitric oxide synthase), *NF-κB* (Nuclear factor kappa B subunit 1), *APOE4* (Apolipoprotein E 4), and *BIN1* (Bridging integrator 1).

Microglia and macrophages, the primary immune cells in the central nervous system, exhibit pronounced sex differences in their responses to neurodegenerative diseases. In healthy brains, microglia show anatomical and developmental differences between sexes, establishing the basis for differential immune responses in disease states. These sex-specific response profiles encompass changes in cytokine expression, metabolic activity, and immunophenotype, as shown in Figure 1.

Several inflammation-related genes are located on the X chromosome, including forkhead box P3 (*FOXP3*), interleukin 2 receptor subunit gamma (*IL2RG*), and toll-like receptor 7 (*TLR7)*, although their interactions with estrogen and aging remain complex and understudied [8,9]. The *FOXP3* gene is associated with chronic diseases. The rs2232365 polymorphism in *FOXP3* has been associated with clinical and pathological features, as well as biomarkers, of HTLV-1 virus infection, specifically in men [10]. Mutations in *IL2RG* cause X-linked severe combined immunodeficiency (X-linked SCID), placing many women at risk of being carriers. These mutations create significant clinical challenges, as bone marrow transplantation—the primary treatment for SCID—carries substantial mortality risk in affected patients [11]. X-linked *TLR7* drives male-biased interferon responses (cytokine response) that worsen demyelination in Alzheimer’s disease. Inhibiting *TLR7* reduces sex differences and protects against tau-induced demyelination and motor impairment in male mice, but not in female mice [12]. Males generally exhibit more pronounced inflammatory responses, as evidenced by the increased number of activated microglia and higher expression of pro-inflammatory genes, such as interleukin 1 beta (*IL1B*) and C-C motif chemokine receptor 2 (*CCR2*), in Y chromosome-intact macrophages [13]. This inflammatory dimorphism is evident in various pathological conditions. In AD models, these sex differences translate into distinct pathological outcomes. Female APP/PS1 mice demonstrate a greater plaque burden and worse spatial learning deficits than males [14]. Similarly, postmortem analysis of human AD brains reveals that male microglia show uniform branching and higher density, while female microglia exhibit diverse morphology and lower density [15]. These differences may explain why women with AD exhibit increased symptom severity and more rapid cognitive decline than men.

The protective effects of estrogen against neuroinflammation are well established in stroke and traumatic brain injury models, where female sex hormones reduce the production of pro-inflammatory cytokines, such as *IL-1β*, tumor necrosis factor-alpha (*TNF*-α), inducible nitric oxide synthase (*NOS*), and nuclear factor kappa B subunit 1 (*NF-κB*) activation [16]. However, this neuroprotection diminishes with aging and menopause, creating a complex picture of AD pathogenesis in women. Early studies have suggested that estrogen-based hormone therapy (HT) provides neuroprotective effects in AD, particularly against Aβ-associated toxicity [17]. However, the Women’s Health Initiative Memory Study (WHIMS)—a large-scale randomized, double-blind, placebo-controlled trial of approximately 4500 women aged 65–79 years—found that HT did not significantly improve cognition compared to placebo in women experiencing age-related cognitive decline [18]. This clinical trial evidence has important implications for treatment recommendations. The lack of cognitive benefit in WHIMS, combined with established cardiovascular and cancer risks associated with HT, means that hormone therapy is not recommended specifically for AD prevention or treatment. The timing hypothesis suggests that estrogen’s neuroprotective effects may be most pronounced during the perimenopausal transition, but initiating HT in older women (as in WHIMS) may be too late to confer meaningful cognitive protection. Additionally, the increased risk of stroke, blood clots, and breast cancer associated with HT outweighs any potential cognitive benefits in this population.

### 3.2. Autophagy Difference

Autophagy, the cellular process responsible for removing damaged components, shows complex sex differences that remain poorly understood, although emerging evidence suggests that these differences appear to be tissue-specific and age-dependent. Several X-linked genes, including *ATP6AP2* and *LAMP2*, participate in autophagy regulation, and mutations in these genes cause autophagy-related diseases [19].

The relationship between sex and autophagy activity appears contradictory across different experimental contexts. Some cellular studies suggest that females may have reduced basal autophagy in certain cell types; for example, males show approximately three-fold higher beclin 1 (BECN1) expression than females in primary human umbilical vein endothelial cells (HUVECs) [20]. However, extrapolating these findings to brain autophagy is problematic because autophagy regulation varies significantly across cell types. Brain autophagy involves diverse cellular populations, including neurons, microglia, astrocytes, and brain macrophages, each with distinct regulatory mechanisms that may respond differently to sex hormones than peripheral endothelial cells. However, clinical evidence suggests a more complex relationship between sex and autophagy. Young female stroke patients demonstrate superior anti-inflammatory responses and better recovery outcomes than males, which is consistent with enhanced rather than impaired autophagy activity. This apparent contradiction highlights the tissue-specific and context-dependent nature of sex differences in autophagy, as autophagy plays crucial roles in neuroprotection, protein clearance, and inflammation resolution after brain injury.

Bioinformatic analysis has revealed that two-thirds of autophagy proteins contain androgen or estrogen receptor binding sites in their promoter regions, indicating potential transcriptional regulation by sex hormones [21]. Although Estrogen receptor 1 (*ER*α) and *ERβ* are transcription factors involved in regulating many complex physiological processes [22], the functional significance of these binding sites in autophagy remains unclear. The presence of hormone receptor-binding sites does not predict whether the regulatory effect will be activating or inhibiting, as transcriptional outcomes depend on complex interactions between tissue-specific transcriptional factors, co-activators, co-repressors, and hormone-receptor complexes. The actual regulatory effects must be validated using experimental approaches, such as chromatin immunoprecipitation followed by sequencing (ChIP-seq). These effects likely depend on tissue type, hormonal context, chromatin state, and specific complement of co-regulatory factors present, contributing to the complex, context-dependent nature of sex differences in autophagy. This area remains critically understudied, representing a significant knowledge gap in understanding the sex-specific mechanisms of neurodegeneration.

### 3.3. Metabolism Difference

Metabolic dysregulation is a central mechanism of aging and neurodegeneration, with distinct patterns emerging between males and females. This dysregulation manifests through two primary pathways: bioenergetic defects, in which mitochondrial dysfunction impairs neuronal energy production, leading to cell death, and biochemical alterations, characterized by protein misfolding and Aβ aggregation [23]. Paradoxically, aged female brains demonstrate a “younger” metabolic profile compared to male brains of the same chronological age, as determined by analyzing aspects such as glucose utilization, oxygen consumption, and cerebral blood flow measurements [24]. Women also maintain superior antioxidant defenses, generating higher levels of protective enzymes while producing lower levels of damaging molecules, including hydrogen peroxide, *NADPH* oxidase, and homocysteine, than age-matched men [25]. These metabolic advantages may explain why women with mild-to-moderate Alzheimer’s Disease (AD) sometimes exhibit cognitive advantages over men at the same stages. However, this protection is not sustained as disease severity progresses [26].

Despite these early advantages, women ultimately face greater metabolic vulnerability to AD. Brain glucose hypometabolism, a hallmark of AD that correlates with disease severity [27], may disproportionately affect women due to the dramatic decline in sex hormones that regulate energetic metabolism during menopause. In addition, animal studies have revealed that age-induced metabolic remodeling occurs earlier in females than in males. In wild-type mice, 44% of genes showed expression changes in females between 6 and 9 months of age, compared to only 5% in age-matched males [28]. Critically, the altered genes in females were predominantly downregulated, with approximately half involving energy metabolism pathways, suggesting an accelerated metabolic decline in the female brain. Notably, microglia appear to mediate sex-specific metabolic responses in patients with AD. Female microglia from APP/PS1 mice shift toward glycolysis in the presence of amyloid plaques, while male microglia maintain their metabolic profile [15]. This sex-specific microglial response may contribute to differential disease progression patterns between males and females.

Estrogen serves as a master regulator of brain metabolism by modulating glycolysis, the tricarboxylic acid cycle, and mitochondrial respiration. The decline in estrogen levels during menopause parallels increased oxidative stress in female brains [23]. Pre-menopausal women maintain superior antioxidant defenses and lower oxidative stress than men, which is largely attributed to the protective effects of estrogen. Estrogen directly promotes mitochondrial biogenesis and enhances mitochondrial respiration in both neurons and glia [7], providing neuroprotection through multiple metabolic mechanisms.

## 4. Genetic Susceptibility to AD

Several genetic factors help explain why women may be more susceptible to AD, despite the generally protective effects of estrogen. The apolipoprotein E4 (*APOE4*) genotype interacts differentially with sex, causing more severe AD pathology in females than in males, with estrogen potentially enhancing rather than mitigating *APOE4*’s detrimental effects. In 5XFAD mice, *ApoE4* females demonstrate reduced microglial plaque coverage despite a paradoxically higher amyloid burden, suggesting impaired clearance mechanisms that may be specific to the female brain environment [7]. The *APOE* gene, primarily known for its role in lipid transport and cholesterol homeostasis, significantly influences brain metabolism differently in males and females. *APOE4* carriers show impaired glucose metabolism and reduced mitochondrial function; however, these effects are more pronounced in women [29].

The bridging integrator 1 (*BIN1*) gene is the second most significant AD susceptibility gene after *APOE* and is involved in complex, sex-specific mechanisms in AD pathogenesis. *BIN1* has at least 15 different known isoforms, with the longest isoform (iso1) being brain-specific, while smaller isoforms like iso9 are expressed ubiquitously [30]. In the adult human brain, *BIN1* is mainly expressed by oligodendrocytes, microglial cells, and glutamatergic neurons, with overall expression reduced in patients with AD compared to healthy individuals [31]. Population genetics studies have shown that *BIN1* variants confer greater AD risk in females and affect pathogenesis through tau pathway dysregulation [32]. However, experimental work has demonstrated that microglial *BIN1* deletion reduces tau spreading specifically in males. These seemingly contradictory findings suggest that *BIN1* functions differently across brain cell types and sexes, potentially acting protectively in microglia while contributing to pathology through other cellular mechanisms. This complexity underscores that the relationship between genetic risk factors and sex-specific AD susceptibility extends beyond simple neuroprotective models.

*TREM2* (triggering receptor expressed on myeloid cells 2) mutations, particularly the R47H variant, confer a significant risk of AD with pronounced sex-specific effects. The R47H variant increases AD risk with odds ratios of 5.05–7.40, comparable to that of *APOE4* heterozygosity. Mechanistic studies have revealed striking sex differences: the R47H mutation exacerbates tauopathy-induced spatial memory deficits and pro-inflammatory transcriptomic changes specifically in female mice, with no effect in males [7]. These mutations impair microglial function and amyloid clearance, with microglial *Trem2* expression being highest in male *ApoE3* mice and reduced by both the *ApoE4* genotype and female sex [33]. In a triple-transgenic model combining *APOE4*, Trem2R47H, and human Aβ pathology, a high-fat diet accelerated early onset LOAD (late-onset Alzheimer’s disease) pathology by 18 months, with females showing greater vulnerability [34]. Pathway analysis revealed sex-specific molecular signatures: upregulated protein processing pathways in 12-month-old males versus downregulated *MAPK* signaling in age-matched females, suggesting fundamentally different cellular stress responses between sexes in this AD model.

## 5. Gender Differences in miRNA Expression

MicroRNAs (miRNAs) are small non-coding RNA molecules that play crucial roles in post-transcriptional gene regulation, with approximately 70% of miRNAs expressed in the brain and 60% of genes regulated by one or multiple miRNAs [35]. Each miRNA can target several hundred genes, making them powerful regulators of cellular processes. To date, more than 2,500 unique human mature miRNAs have been identified (miRBase v22). Understanding sex differences in miRNA expression and response has important implications for assessing neurological disease risk, including Alzheimer’s disease (AD), and for developing targeted therapeutic intervention strategies in both basic research and clinical settings.

Sex differences in miRNA expression arise through two primary mechanisms. First, sex hormones, particularly estrogen and progesterone, directly influence miRNA expression patterns. Second, the X chromosome harbors a disproportionately high number of brain-expressed miRNAs compared to other chromosomes [36]. These X-linked miRNAs are predominantly organized in clusters, including miR-532/188, miR-221/222, miR-98/Let7f, and miR-363/106a/20b/92a [37], positioning them as potential biomarker candidates for the early detection of AD in females.

miRNAs play critical roles in inflammatory processes, with notable sex-specific patterns across different disease contexts. In rheumatoid arthritis (RA), a female-predominant disease similar to AD, elevated intracellular levels of miR-19 exacerbate the production of pro-inflammatory interleukin 8 (*IL-8*) and *IL-6* by primary fibroblast-like synoviocytes. Additionally, miR-18 contributes to a positive feedback loop that amplifies *NF-κB*-driven inflammation in RA synoviocytes. Importantly, both miR-18 and miR-19 are encoded not only on chromosome 13 within the miR-17/92 cluster but also within another miRNA cluster on the X chromosome. This dual chromosomal location directly contributes to higher intracellular concentrations of these miRNAs in female patients, potentially explaining the female predominance of RA [38]. Sex differences in miRNA expression have also been observed in other inflammatory conditions. In a chronic myocarditis mouse model following CVB3 infection, Let-7a expression was downregulated in male hearts, while Let-7b expression was upregulated in female hearts [39], showing significant sex differences in this infection. Similarly, in LPS (lipopolysaccharide)-stimulated microglia, miRNA responses were more pronounced in cells from female mice, particularly for miR-16-5p, miR-99a-5p, and miR-191-5p [40]. Collectively, these findings demonstrate that inflammatory miRNA responses exhibit consistent sex-specific patterns across different organ systems and disease models.

The therapeutic potential of miRNA modulation shows significant sex- and age-dependencies that must be considered in treatment development. In stroke models using miR-15a/16-1 antagomir treatment, young female mice demonstrated superior survival and sensorimotor function compared to young male mice [41], emphasizing the importance of considering both sex and age when developing miRNA-based therapeutic strategies. In middle-aged female rats, experimental stroke caused significant sensory motor deficits in both sexes. However, miR-20a-3p treatment only ameliorated cognitive impairment in female animals three months after stroke [42]. Conversely, some miRNA effects appear to be sex-independent. MiR-34a overexpression induces rapid cognitive impairment associated with intracellular Aβ accumulation and tau hyperphosphorylation across multiple brain regions, without apparent sex differences. This phenomenon occurs by targeting *ADAM10*, *NMDAR 2B*, and *SIRT1* RNAs, which are profoundly reduced by miR-34a overexpression. These findings suggest that miR-34a-mediated cognitive decline may represent a polygenic risk factor model for late-onset Alzheimer’s disease (LOAD) [43].

Several miRNAs show significant associations with Aβ levels, although not all demonstrate sex-specific patterns. Concentrations of miR-15b were significantly lower in Aβ-PET (positron emission tomography)-positive individuals than in Aβ-PET-negative individuals [44]. Furthermore, plasma concentrations of miR-125b and miR-125-5p time interaction were negatively associated with regional Aβ-PET standard uptake value ratios in the right anterior cingulate cortex. These findings suggest an anti-Aβ effect of miR-15b and indicate a biological link between miR-125b and Aβ-independent neurotoxic pathways. Plasma concentrations of miR-125b and miR-125-5p may serve as potential biomarker candidates for predicting disease progression, although no associations with sex or age were identified in these studies. While comprehensive reviews have summarized miRNAs that directly regulate AD pathology—including the expression, phosphorylation, and alternative splicing of Aβ and tau, as well as related regulatory kinases [45]—systematic investigations of sex-specific associations with these regulatory mechanisms remain limited and represent an important area for future research.

## 6. MiRNA Biomarker Candidates for AD in Women

### 6.1. miRAN Advantages as Biomarker Candidates for AD

Currently, Alzheimer’s disease (AD) diagnosis relies primarily on five classes of biomarkers, including neuroimaging biomarkers from CT and MRI, cognitive behavior tests MMSE (the Mini-Mental State Examination) and MoCA (the Montreal Cognitive Assessment), and the three categories within the ATN framework. The National Institute on Aging–Alzheimer’s Association (NIA-AA) formulated a research framework known as the ATN classification system, which categorizes biomarkers based on amyloid-β (Aβ) deposition (A), pathologic tau (T), and neurodegeneration or neuronal injury (N). However, cognitive tests cannot identify presymptomatic or prodromal stages. Cerebrospinal fluid (CSF) biomarkers, including Aβ42, total tau, and phosphorylated tau (p-tau181, p-tau217), are established protein markers but require invasive lumbar puncture procedures. Recent advances in plasma biomarkers, particularly p-tau217 and p-tau181 [46,47], offer improved accessibility but still face challenges in early-stage detection and sex-specific validation. While PET imaging provides non-invasive visualization of amyloid and tau pathology, it primarily detects advanced pathological changes, is costly, and has limited availability for population screening. Furthermore, current biomarker approaches lack systematic validation for sex-specific differences in expression and diagnostic accuracy. Blood-based miRNA biomarkers could address these limitations by detecting molecular alterations during the prodromal stages while enabling sex-specific diagnostic optimization, capabilities that current clinical approaches do not adequately provide. Within the ATN framework, miRNA biomarkers would be classified under the N category as early molecular indicators of neuronal injury and dysfunction.

As biomarker candidates, miRNAs offer several key advantages, particularly their remarkable stability and ability to cross the blood-brain barrier (BBB). Regarding stability, miRNAs demonstrate exceptional resistance to degradation in biological samples. A recent study demonstrated that miRNAs remain stable in serum samples stored at −20 °C for up to 3 months (avoiding repeated freeze-thaw cycles), with protective agents extending storage time even at room temperature (25 °C), meeting practical clinical requirements [48]. Importantly, miRNA levels are unaffected by food intake, with no significant differences observed between fasting morning samples and post-meal samples, offering practical advantages over traditional biomarkers that require fasting conditions [49].

The blood-brain barrier (BBB), an essential component of the neurovascular unit that controls molecular exchange between the blood and brain, can be traversed by miRNA-containing extracellular vesicles that carry neuronal information. Four exosomal miRNAs (miR-127-3p, miR-423-3p, miR-378b, and miR-106-3p) showed correlated differential expression across brain tissue, cerebrospinal fluid, and plasma, demonstrating the potential of miRNAs to serve as liquid brain biopsies [50]. Using minimal blood volumes (0.2 mL), miRNAs with a coefficient of variation (CV) of less than 0.20 provided reliable measurements [51]. These brain-derived extracellular vesicle miRNAs carry neuronal information and show promise as early biomarkers in preclinical individuals. Functional analysis revealed the enrichment of miRNA target genes in neurodegenerative pathways, highlighting synuclein alpha (*SNCA*), cytochrome c somatic (*CYCS*), and microtubule-associated protein tau (*MAPT*) as key therapeutic targets.

### 6.2. Sex-Specific miRNA Biomarkers for AD in Females

Alzheimer’s disease in females has attracted increasing attention due to the larger affected populations and sex-specific pathological features, highlighting the need for female-specific biomarkers. Understanding sex-based miRNA expression patterns provides crucial insights into the dysregulation of multiple biological processes associated with the disease. MiRNAs influence AD pathogenesis and pathology through their roles in Aβ metabolism, tau function, and immune-inflammatory responses, making them promising biomarker candidates. However, compared with non-sex-related biomarker studies, research on biomarkers specifically for female patients with AD remains in its early stages.

Llera-Oyola et al. conducted a bioinformatics analysis of six GEO datasets from AD brain tissues and blood samples, revealing distinct miRNA expression profiles in female patients [52]. In AD brain samples, five miRNAs were significantly downregulated, while miR-105-3p was significantly upregulated, as shown in Table 1. More importantly, for clinical applications, seven miRNAs were upregulated in the blood samples of female patients with AD (Table 1), with an additional eight miRNAs showing upregulation in the blood samples of both female and male patients with AD. The seven female-specific upregulated miRNAs—miR-296-5p, miR-766-3p, miR-1304-3p, miR-4326, miR-4685-3p, miR-let-7d-3p, and miR-671-3p—demonstrate particular promise as AD circulation biomarkers due to their upregulation in easily accessible blood samples, highlighting their potential for non-invasive diagnostic testing. After comprehensive research, we found that miR-296-5p, which is derived from plasma vesicles, could potentially be utilized as a biomarker candidate for AD patients [53]. Additionally, miR-766-3p is significantly upregulated in plasma exosomes from the elderly, which is also related to autophagy and may serve as a promising biomarker for brain aging [54]. In addition, only miR-296-5p overlapped with previous studies on sex-biased miRNAs [55], which used public datasets and literature to retrieve sex hormone-related miRNAs.

The sex-specific nature of AD extends to region-specific miRNA expression patterns, which have important implications for biomarker development. Neuroinflammation shows distinct regional patterns in women, with the highest levels observed in the subiculum. Statistical significance was observed only in female patients. Four inflammatory miRNAs—miR-146a, miR-34a, miR-125b, and miR-155-5p—showed increased expression specifically in the parietal cortex of female patients with AD, as detailed in Table 1. This regional specificity is supported by neuroimaging studies using in vitro autoradiography of the hippocampus, entorhinal cortex, and parietal cortex with neuroimaging tracers PK1195 and T807. These studies revealed region-dependent elevations of translocator protein (TSPO) density in AD relative to cognitively normal and mild cognitive impairment (MCI) patients, but only in women. Although tau deposition was highest in the entorhinal cortex and significant in both sexes, the density of tau and TSPO showed a positive and significant correlation only in women with AD [56]. These findings suggest that the expression levels of these four miRNAs may serve as indicators of cognitive status in females with AD, potentially providing sex-specific biomarkers for disease monitoring and therapeutic response assessment.

**Table 1:** MiRNA Biomarker candidates in female patients with AD.

MiRNA Names	Up- or Down-Regulated	Tissue Types	Sample Size (AD/CON)	Reference
MiR-431-3P, -494, -653, and -668. MiR-105-3p	Downregulated Upregulated	Tissue Tissue	15/12	[52,57,58,59,60]
miR-296-5p, -766-3p, -1304-3p, -4326, -4685-3p, let-7d-3p, and -671-3p	Upregulated	Blood	739/148	[52,61,62]
MiR-146a, -34a, -125b, and -155-5p	Upregulated	Tissue	18/18	[56]
MiR-642-5p	Upregulated	Tissue	460 f/188 m *	[63]
MiR-146b-5p, -150-5p, -342-3p	Upregulated	CSF	28 f/28 m *	[64]

* f: female; m: male.

Vattathil et al. conducted sex-stratified analyses of miRNAs and identified five miRNAs from a total of 528 that were associated with cognitive trajectories [63] These miRNAs exhibited sex-biased differential expression in one or more Alzheimer’s disease (AD) endophenotypes. The effects were consistent in both males and females for all miRNAs, except for miR-642a-3p/5p, which demonstrated opposite effects on working memory between sexes. Consequently, miR-642a-3p/5p is proposed as a biomarker candidate for female patients with AD, as shown in Table 1. Furthermore, their Mendelian randomization (MR) studies indicated that the identified miRNAs, single-nucleotide polymorphisms (SNPs), and AD endophenotype observations support a model in which miRNA expression has a causal effect on the AD endophenotype. Notably, these differentially expressed miRNAs provided evidence consistent with a causal role, setting the stage for future mechanistic studies of miRNAs in AD and its endophenotypes. In addition, Sandau et al. assessed the effects of sex differences on miRNA expression in cerebrospinal fluid (CSF) extracellular vesicles (EVs) in AD [64]. They found that three miRNAs (miR-146b-5p, miR-150-5p, and miR-342-3p) were significantly elevated by at least 1.5-fold in females compared to males, as shown in Table 1.

## 7. Discussion

This review explored sex-driven differences in biological mechanisms, miRNA expression patterns, and miRNA biomarker candidates for female patients with AD. Gender differences mediated by sex hormones and X chromosome inheritance contribute to distinct pathological features beyond the well-established sex differences in lifespan and disease prevalence, including altered inflammatory responses, metabolic dysfunction, and autophagy impairment. Through comprehensive analysis of the available literature, we identified altered miRNA candidates across multiple sample types—brain tissue, blood, and cerebrospinal fluid—with approximately 20 miRNA candidates emerging from studies encompassing more than 1000 patients. These findings provide a foundation for future investigations of sex-specific AD biomarkers.

The implications of this research extend to personalized medicine approaches and the exploration of novel therapeutic targets. Genomic analysis has become widely accepted and frequently utilized in clinical practice for patients with AD, including *APOE3* and *APOE4* genotyping in specific populations. However, these genetic variants also have sex-specific effects on disease manifestation. For instance, one study demonstrated that Let-7d-5p expression levels were significantly increased in AD *APOE3* homozygous females compared with healthy control *APOE3/4* heterozygous females [64], suggesting that both genotype and sex influence miRNA expression levels and AD pathology. Let-7d-5p affects inflammatory signaling pathways and can alleviate inflammatory responses in microglia by targeting *MAP3K1* and inactivating *ERK/p38 MAPK* signaling [65], making it a potential therapeutic target. While genotype testing alone provides valuable clinical information, incorporating sex-specific analyses could significantly broaden and deepen the clinical utility of genetic assessments for AD.

Research in this area remains significantly understudied, representing a critical gap in our understanding of the pathogenesis and diagnosis of AD. Despite the promising identification of approximately 20 miRNAs in diverse patient populations and sample types, the limited overlap between studies underscores several important limitations. Current miRNA biomarker research faces significant methodological challenges, including small sample sizes, lack of standardized protocols for sample collection and processing, heterogeneous analytical platforms, and limited replication across independent cohorts. Furthermore, the majority of studies have not systematically examined sex-specific differences, and many identified miRNA candidates lack functional validation to establish their causal role in AD pathogenesis. The heterogeneity observed in study populations, sample collection methods, experimental protocols, and analytical platforms likely contributes to the variability in findings across investigations. These limitations collectively emphasize that female-specific miRNA biomarker research for AD remains in its early discovery phase, requiring substantial investment in replication and validation efforts before clinical translation can be achieved. 

Therefore, several key priorities must be addressed to advance this field. Large-scale multi-center studies with standardized protocols for sample collection, processing, and analysis are essential for generating reproducible and clinically relevant findings. Additionally, functional validation studies are needed to establish causal relationships between identified miRNAs and AD pathology in females. Although the Let-7d-5p example demonstrates the feasibility of such investigations, further studies with larger sample sizes and additional miRNA candidates are required to advance our understanding. Furthermore, integration with other omics data, including SNPs and protein expression profiles, along with established biomarkers, will be crucial for developing comprehensive diagnostic panels that can accurately predict and monitor AD progression in female patients.

## 8. Conclusions

This review shows that while female-specific miRNA biomarkers for AD represent an emerging research direction, this field remains in its early stages. The identification of approximately 20 miRNA candidates across different sample types should be interpreted as early proof-of-concept evidence rather than as established sex-specific biomarkers. The current state of the field is characterized by significant methodological heterogeneity, limited sample sizes, and a lack of consensus across independent studies, underscoring the fact that this research remains in its infancy. The lack of replication across studies and the absence of standardized reference comparisons with established AD biomarkers emphasize that substantial foundational work is still required. However, these candidates derived from the blood and cerebrospinal fluid of female AD patients, which offer accessible targets for future studies and clinical validation. Future progress will depend on large-scale, multi-center validation studies employing standardized protocols, direct comparisons with CSF/plasma protein biomarkers, and rigorous methodological approaches before any clinical translation can be considered. Currently, this field represents a promising research opportunity that requires significant development rather than an established diagnostic approach.

## Abbreviations

The following abbreviations (without full names in the content) are used in this manuscript:

MAPK	Mitogen-activated protein kinase
NADPH	Nicotinamide adenine dinucleotide phosphate
MAP3K1	Mitogen-activated protein kinase kinase kinase 1
ERK/p38 MAPK	Mitogen-activated protein kinase 1
APP/PS1	Amyloid beta precursor protein/Presenilin 1
5xFAD	Five familial Alzheimer’s disease mutations
ATP6AP2	ATPase H+ transporting accessory protein 2
LAMP2	Lysosomal-associated membrane protein 2
ADAM10	Metallopeptidase domain 10
LPS	Lipopolysaccharide
NMDAR2B	Glutamate Ionotropic receptor NMDA Type subunit 2B
SIRT1	Sirtuin 1
HTLV-1	Human T-lymphotropic virus type 1
CVB3	Coxsackievirus B3
